# AVO reflectivity and pre-stack seismic impedance inversion for gas sand channel detection at South Abu El Naga Field, Onshore Nile Delta, Egypt

**DOI:** 10.1038/s41598-025-04251-6

**Published:** 2025-06-20

**Authors:** Nourhan Ahmed Al-Ashqar, Abdel-Khalek El-Werr, Ahmad Sobhy Helaly, Azza Kamel

**Affiliations:** 1 Geophysics Department, Faculty of Science, Ain Shams University, Cairo, Egypt; 2 Applied Geophysics (Seismic Methods), Geophysics Department, Faculty of Science, Ain Shams University, Cairo, Egypt; 3 Applied Geophysics (Potential Methods), Geophysics Department, Faculty of Science, Ain Shams University, Cairo, Egypt; 4 Exploration and Board Member, El-Wastani Petroleum Company (WASCO), Plot No. 188, El Tesaeen St., Fifth Settlement, New Cairo, Egypt

**Keywords:** AVO reflectivity, Impedance, Gas reservoir, Nile delta, Geology, Geophysics

## Abstract

The reservoir compartment is a major uncertainty at South Abu El Naga gas field, onshore Nile Delta, Egypt. The objective of this study is to detect Abu Madi gas sand reservoir using different pre-stack inversion techniques such as AVO reflectivity attributes, impedance methods, and lambda-mu-rho (LMR) analysis. The Messinian sandstone gas reservoir at the study area was effectively characterized using these three techniques. Well logs, 2D partial angle stack, and full angle stack seismic sections are the available dataset used to derive several seismic pre-stack inversion attributes. The results of these attributes show that the gas sand bodies are clearly separated from shale and detect the gas channel lateral edges from the cutting mud filled channel. These findings determine the utility of integrating AVO reflectivity attributes and impedance methods in enhancing geophysical interpretation, reducing uncertainty, aiding exploration and support more accurate compartment delineation in data-limited settings and provide a convenient workflow applicable to other areas facing similar exploration and challenges.

## Introduction

**Fig. 1:**
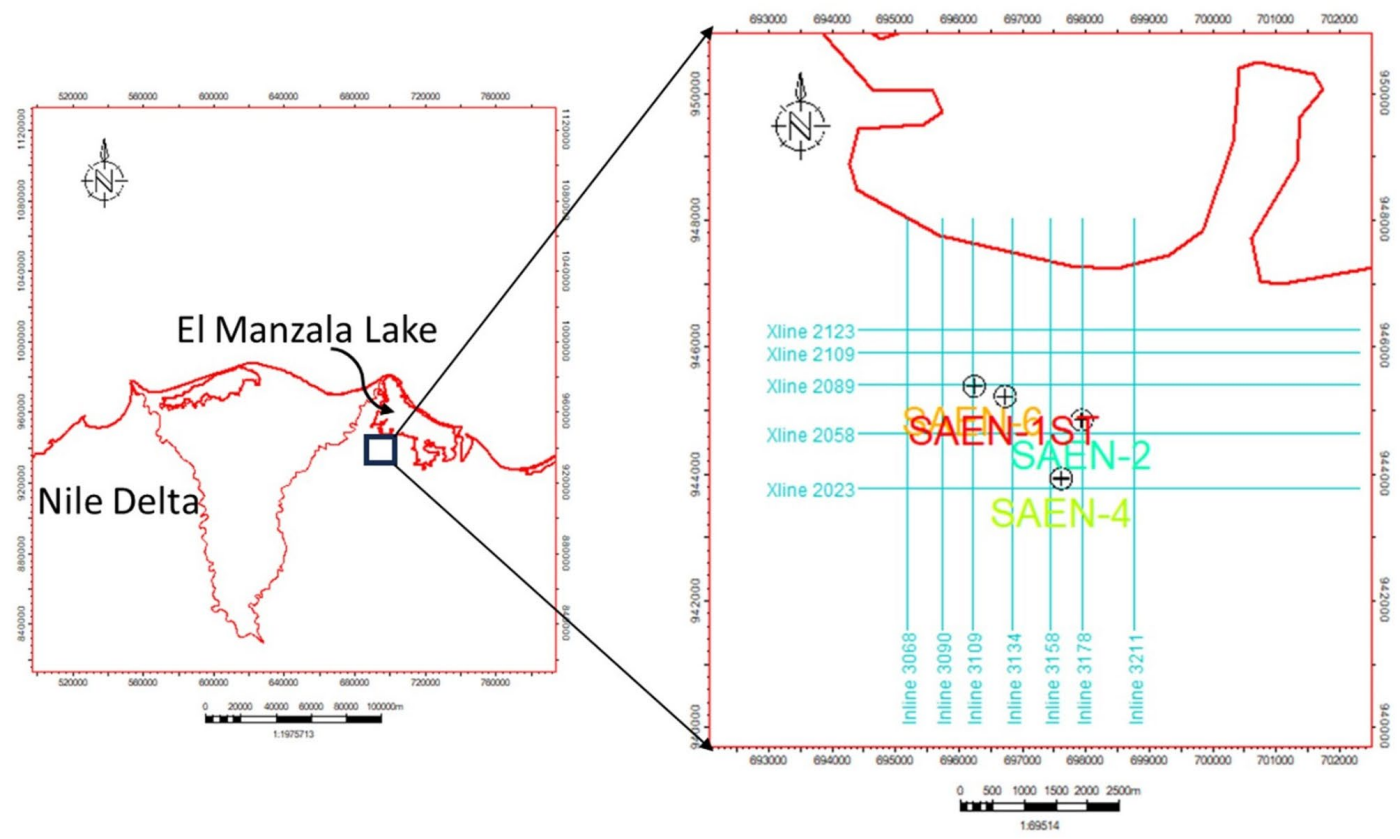
Location map of the West El Manzala concession study area and Base map of the four wells and the 2D-seismic lines, created using Petrel (2019) software, Petrel subsurface software | SLB.

The study area is South Abu El Naga gas field, a geologically complex zone with proven hydrocarbon systems accommodated with clastic reservoirs. These reservoirs are typically influenced by faulting and stratigraphic variability, making gas sand detection particularly challenging. South Abu El Naga gas field located in West El Manzala concession at Onshore East Nile Delta, Egypt, west to southwest of El Manzala Lake (northeastern part of the Nile Delta), between latitudes 31° 16′ 35″ N and 31° 17′ 23″ N and longitudes 31° 36′ 05″ E and 31° 37′ 36″ E as shown in Fig. 1.

The Nile Delta is a giant gas province because its discoveries represent about 82% of all Egyptian gas discoveries with reserves of 58 TCF^1^. The main gas-bearing reservoirs in the Nile Delta province are developed in the sandstone interbeds of the late Miocene age (Messinian) of Abu Madi Formation^2–4^. A huge gas volumes were produced from the Miocene and Pliocene Sandstone reservoirs hence; proper characterization is significant for reducing drilling risks and improving the recovery of oil and gas^5^.

Several studies and exploration activities have focused on understanding the geological and geophysical characteristics of the Nile Delta region. These studies help in identifying potential hydrocarbon reservoirs and understanding the fault systems that influence hydrocarbon migration and trapping. For instance, studies by Khalil and McClay^6^ and Aboulela^7^ have contributed to the seismic characterization of the region. Research on the stratigraphic sequences of the Nile Delta, including works by Abdel-Fattah and Slatt^8^ and Shehata^9^, has revealed important insights into the depositional environments and historical geological processes. Also, several previous studies in the East Nile Delta have primarily depending on using AVO reflectivity and pre-stack seismic inversion for gas sand delineation including Gobashy et al.^10^ and El-Mowafy^11^.

Dana Gas has announced a new gas discovery in the Nile Delta, Egypt. The discovery well ‘South Abu El Naga-1ST’, located in the West El Manzala Concession, encountered 7.2 and 12.6 m of net pay in the Abu Madi Upper and Lower Formation respectively, and 4.8 m in the Kafr El Sheikh Formation (Egypt Oil and Gas, 2013, Egypt Oil & Gas (egyptoil-gas.com)). South Abu El Naga gas field is part of the larger Nile Delta Basin, which is known for its complex geological structures and rich hydrocarbon potential. The field is characterized by complex geological features, including deltaic deposits, fault systems, and varied stratigraphy. Understanding these complexities is essential for gas sand reservoir identification. Hydrocarbon potential of East Nile Delta Basin make it an economically strategic target for gas exploration. Accurate identification of gas-bearing sands in this area could significantly reduce drilling risk and improve reserve estimates, Saleh et al.^12^.

The seismic data was acquired using symmetrical split-spread type with orthogonal patch length 7750 m (3875 m–25 m–0 m–25 m–3875 m), receiver spacing and shot spacing equal 50 m while, the receiver line and shot line spacing equal 300 m. Nominal Fold is 65 and record length is 6 s. Processing sequence consists of standard steps: Noise attenuation, Surface Consistent Amplitude Compensation, Surface Consistent Deconvolution and, Radon De-multiple.

The available data in South Abu El Naga study area are four well logs and 2D seismic sections. The available well logs are represented by gamma ray, deep resistivity, P-wave velocity (Vp), S-wave velocity (Vs), density (ρ), water saturation (Sw), shale volume (Vsh), and porosity (φ). The available seismic sections are partial angle stacks of near (0°–6°), mid (8°–12°) and far (36°–40°) and full angle stack seismic sections.

The main objective of this study is to detect Abu Madi gas sand reservoir using AVO reflectivity attributes, impedance methods, and lambda-mu-rho (LMR) analysis and assess the ability of these attributes in detecting gas sand within a geologically complex and data-constrained. By integrating these techniques, the study achieves improved discrimination between gas-bearing and wet sands. The novelty of this work lies in its integrated application of AVO analysis and angle-dependent impedance inversion to enhance reservoir detection at South Abu El Naga gas field. This approach contributes a practical methodology for gas sand detection in similar geological settings globally.

## Geologic setting

A comprehensive overview of the structural and stratigraphic settings of the onshore Nile Delta, highlighting its geological complexity and significance in hydrocarbon exploration has been summarized as follows:

**Structural setting:** The onshore Nile Delta is characterized by a complex structural framework that has evolved through various tectonic phases, including rifting, subsidence, and inversion. The Nile Delta’s structural setting has been shaped by the extensional tectonics associated with the opening of the Neo-Tethys Ocean during the Mesozoic era. This extension created numerous normal faults and half-graben structures, which are prominent in the subsurface geology of the delta.

**Fig. 2:**
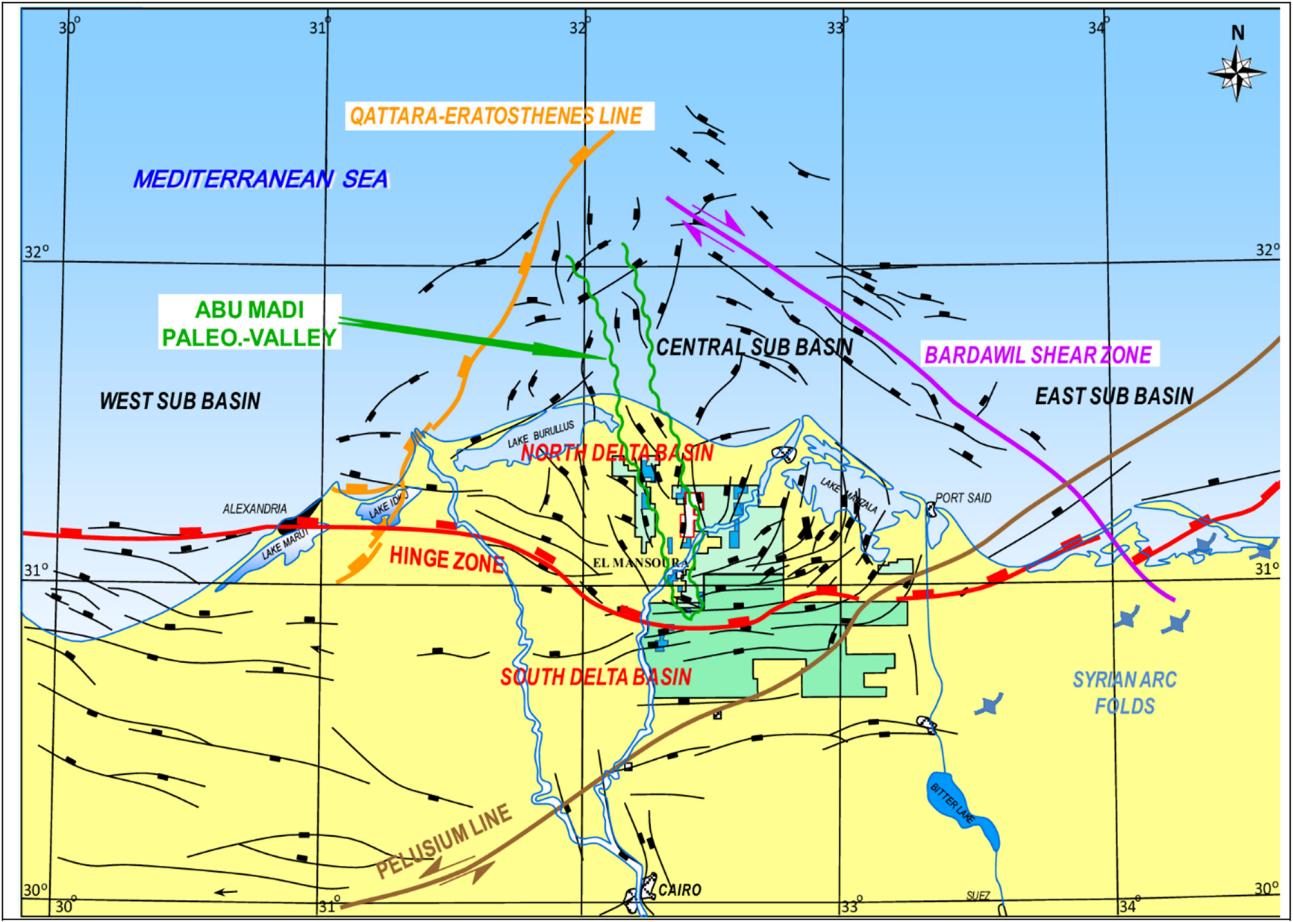
The general structural framework of the Nile Delta Basin sketch map, after^17–19^.

The Nile delta is dissected by three main structural trends, including, the Temsah trend, a northwest–southeast (NW–SE) trend, active during the Miocene, the Rosetta trend, a northeast–southwest (NE-SW) trend of Late Cretaceous age and the Tethyan trend, an east west (E-W) trend, related to the original continental margin rifting of the southeastern Mediterranean during the early Mesozoic^13–15^. These faults have played a significant role in controlling sediment deposition and hydrocarbon migration pathways^16^ Fig. 2 show the general tectonic framework of the Nile Delta.

**Fig. 3:**
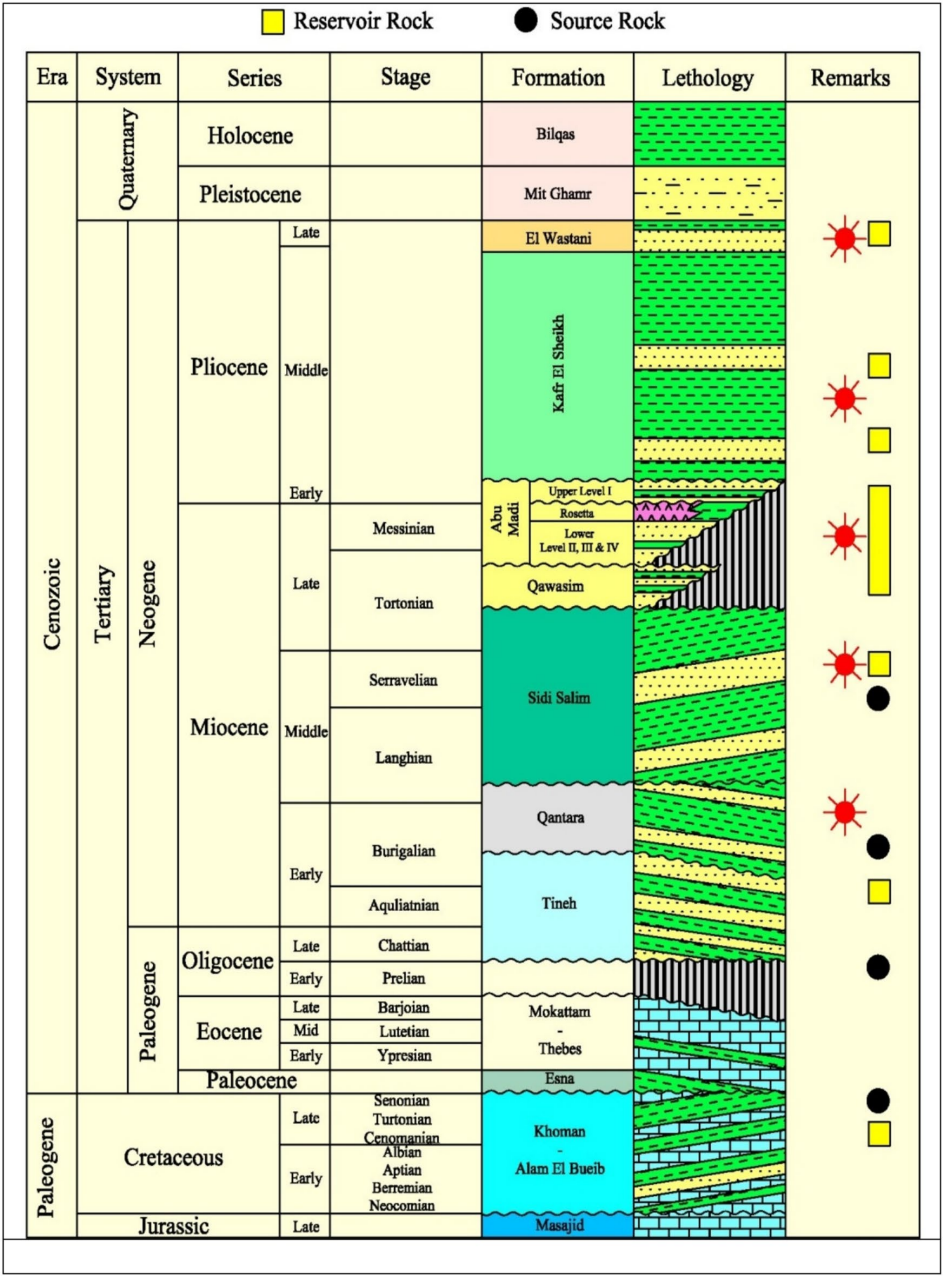
The generalized stratigraphic column of the Nile Delta, after^33^.

**Stratigraphic setting**: The stratigraphy of the Nile Delta is marked by a thick succession of sedimentary deposits that record the interplay between fluvial, deltaic, and marine processes^20^. These deposits extent from the Mesozoic to the Quaternary period. The basal units of the Nile Delta are composed of Mesozoic sedimentary rocks, including sandstones and shales, deposited in a fluvial and shallow marine environment^16^. These formations are often reservoir rocks in hydrocarbon exploration^21^. Overlying the Mesozoic strata are the Paleogene and Neogene deposits, which include thick sequences of marine shales, siltstones, and sandstones^22^. The Oligocene to Miocene Abu Madi Formation, in particular, is a significant hydrocarbon-bearing unit characterized by fluvial to deltaic sandstones^23^. The most recent deposits in the Nile Delta are of Quaternary age, comprising fluvial and deltaic sands, silts, and clays. These sediments have been deposited by the Nile River and its distributaries, forming the modern deltaic plain^24^. The stratigraphic succession in the Nile Delta is marked by several deltaic cycles, reflecting changes in sea level, sediment supply, and tectonic activity. These cycles are represented by alternating sequences of pro-delta shales, delta-front sandstones, and delta-plain silts and clays^25^. Figure 3 shows generalized stratigraphic column at onshore Nile delta.

The Nile Delta’s main reservoir sources range in age from the Oligocene to the Late Pliocene. The potential for probable hydrocarbon accumulations in the Nile Delta was increased by the presence of various mature source rocks (Jurassic to Early Miocene), as well as structural and stratigraphic traps^22,26^. The Jurassic and Upper Cretaceous-Lower Paleogene source rock intervals were the main sources of the thermogenic gas, condensate, and light oil accumulations in the Nile Delta^27^. The major hydrocarbon exploration targets, particularly in the onshore region, are different depositional facies (fluvial and estuarine) found in the Upper Messinian sequence of the Nile Delta; Abu Madi Formation^23,28^. The quality of the Abu Madi reservoir facies is largely controlled by their initial depositional conditions, whereas the post-depositional attributes play a relatively minor role^29,30^

The clastic deposits of the Nile Delta are composed of three main sedimentary cycles. Miocene cycle, whose base is rarely recorded in the delta region, and it is mostly composed non-marine to shallow marine deposits of the Sidi Salim, Qawasim and Abu Madi formations^22,31^. This major sea level fall caused the Mediterranean Sea’s water level to drop significantly, which led to extensive erosion; the Mediterranean region was the place of the formation of enormous canyon incisions, and the development of the deposition core of the basin contains salt deposits^32^. Plio-Pleistocene cycle comprising the open marine Kafr El Sheikh Formation and the deltaic El Wastani, Mit Ghamr and, Bilqas formations. Holocene cycle comprises the top of the section.

## Methodology

Seismic interpretation of the available seismic sections and well logs is the **first step** of the workflow focused to understand the different structures of Abu Madi levels in the study area and to identify the sand channel distribution by extracted seismic attributes.

Then, the workflow **second step** includes two main stages; the **AVO reflectivity** attributes methods and **pre-stack inversion impedance** methods. AVO reflectivity attributes were carried out to produce intercept (A), gradient (B), AVO product (A*B) and scaled poisson’s ratio (A + B) while, Pre-stack simultaneous inversion was applied to produce elastic parameters such as P-impedance (Zp) and S-impedance (Zs) to recognize lithology and to detect gas sand channel compartments. A gas-bearing reservoir channel can cause an obvious strong contrast in P-impedance, S-impedance and density to effectively discriminate gas-bearing sand within Abu Madi Formation in South Abu El Naga field. The resulted sections were used to better define the reservoir.

In seismic refection, the amplitude character generally varies with offsets (AVO) or the change of the incidence angle (AVA). AVO method allows better determination and evaluation of reservoir rock properties and fluid content, depending on the velocity, density, and Poisson’s ratio contrast^34^. Knott^35^ and Zoeppritz^31,36^ developed the theoretical basis for the AVO theory. They developed the equations for plane-wave refection amplitudes as a function of the incidence angle. Bortfeld^37^ simplified Zoeppritz’s equations to understand how the refection amplitudes vary or depend on the incidence angle and physical parameters.

There is a linear relationship between the amplitude and sine squared angle of incidence Eq. (1)^38,39^. The intercept is the amplitude at zero offset/angle, and the gradient is, the slope or function of compressional and shear wave velocities and reflection coefficients, in addition to density contrast and these are useful in locating gas reservoirs. The intercept and gradient are related to the elastic properties of the ground^40^.

1 $$R\left( \theta \right) = A + B\sin^{2} \theta$$

The AVO attributes can identify and distinguish between the change in lithology and fluid content by the anomalies that obtained from the AVO analysis based on the calculated two parameters Intercept A and Gradient B of Aki-Richards (1980) equation^41^. AVO reflectivity attributes, such as the intercept (A), gradient (B) and their combinations; like AVO product (A*B), Scaled Poisson ratio change (A + B), shear reflectivity (A-B) are produced directly from pre-stack seismic gathers.

Using the partial angle stacks of near (0°–6°), mid (8°–12°) and far (36°–40°) with proper wavelet, the results of the pre-stack are the P-impedance (acoustic impedance Zp = ρVP), S-impedance (shear impedance Zs = ρVS), density and the ratio of compressional and shear-wave velocities Vp/Vs ratio. The P-impedance and Vp gave information on the nature of the fluid while, the S-impedance and Vs are considered as a lithological indicator, because S-wave cannot pass through liquids^42^.

Following Goodway^43^ work the Lamé parameters of lambda-rho (λρ) and mu-rho (μρ) attributes were estimated further as λ is described as the most sensitive fluid indicator. The interpretation of the lambda (λ) and mu (μ) attributes is: The λ term, or incompressibility, is sensitive to pore fluid, whereas the μ term, or rigidity, is sensitive to the rock matrix. The Lamé parameters were obtained during the simultaneous inversion from Zp and Zs.

Goodway^43^ proposed a new approach to AVO inversion based on the Lamé parameters λ and μ, and density ρ, or Lambda-Mu-Rho (LMR).

2 $$\mu \rho = Z_{S}^{2}$$

3 $$\lambda \rho = Z_{p}^{2} - 2Z_{s}^{2}$$

Equation (2 and 3) shows Lambda-Mu-Rho (LMR) calculations, where: *Z*_*p*_ is P wave impedance and *Zs* is Shear wave impedance.

One of the significant challenges in seismic interpretation is the ambiguity from seismic attributes. Seismic attributes are often non-unique, meaning that similar seismic responses can result from different geological conditions, making interpretation highly uncertain without sufficient geological setting^44^. For example, in complex geological environments such as the Nile Delta, impedance contrasts may be interpreted as indications of hydrocarbons, when in fact they are due to lithological or depositional variations. This interpretation ambiguity is combined when the data quality is low. To address this challenge, it is essential to integrate AVO seismic attributes and inversion to reduce interpretation uncertainty and improve the reliability of seismic insights.

Seismic inversion, despite being a powerful tool for subsurface characterization, is highly sensitive to various sources of uncertainty. The accuracy of inversion results is dependent on the quality of the seismic data. Low-frequency models, often derived from well log data, can introduce significant errors if poorly constrained, leading to inaccurate impedance estimates and potentially misleading interpretations of subsurface properties^45^. Additionally, noise sensitivity is a major limitation in seismic inversion, as random noise, acquisition footprint, or multiple reflections can distort the amplitude data.

AVO analysis assesses variations in seismic reflection amplitudes with offset, aiding in the identification of fluid content and lithology. In the Nile Delta, AVO analysis has been instrumental in differentiating gas-bearing sands from dry sands. Seismic inversion, which converts seismic data into quantitative rock properties, matches AVO by offering detailed subsurface models. Integrating AVO inversion and seismic attributes enabled delineation of gas sand and reduce exploration risks. However, challenges such as poor near-offset data and complex lithological variations can affect the reliability of the analysis.

## Results

1. Seismic interpretation.

Twelve 2D seismic sections were interpreted using the Petrel™ (version 2019) software developed by Schlumberger, illustrating the two geologic horizon tops and their dissected faults. These geologic formation tops were identified by well-to-seismic tie of SAEN-2 well and arranged from young to old as follows: Upper Abu Madi and Lower Abu Madi.

**Fig. 4:**
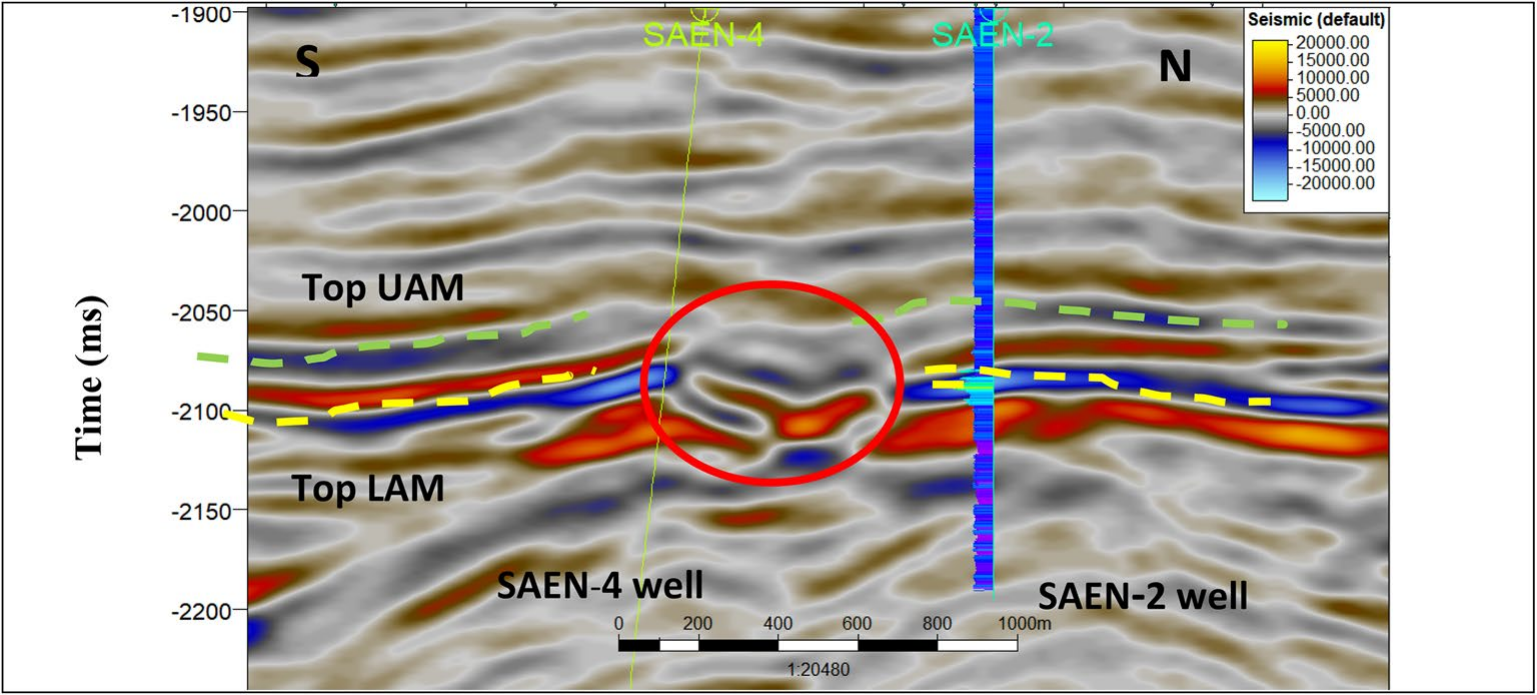
N-S 2D seismic 3178 inline section shows the gas sand reservoir compartments and the mud filled cutting channel, where the target tops characterized by bright amplitude events at 2570 and 2100 ms.

**Fig. 5:**
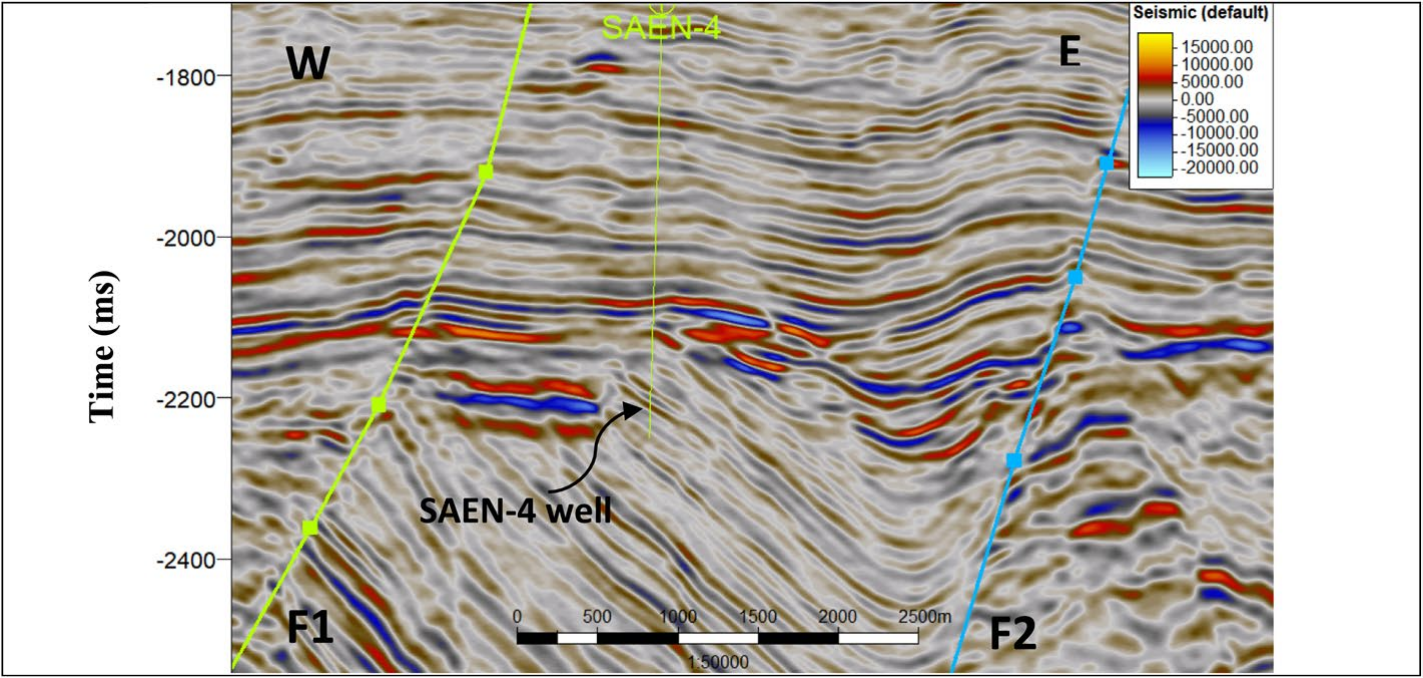
E-W 2D seismic 2023 crossline section passing by the study area shows SAEN 2 and SAEN 4 wells and the two main faults in the study area.

Figure 4 shows a 2D seismic 3178 inline trending N-S, passing through SAEN-2 and SAEN-4 wells. It shows two tops of Upper Abu Madi and Lower Abu Madi Formation. It also shows compartment gas sand channel of the Lower Abu Madi Formation crossed by a very clear mud filled cutting channel in its central part shown in red color. Figure 5 shows a 2D seismic 2023 crossline trending E-W, passing through SAEN-4 well and manifesting two main step-like normal faults.

2. Pre-stack inversion.

2.A) AVO reflectivity attributes method.

**Fig. 6:**
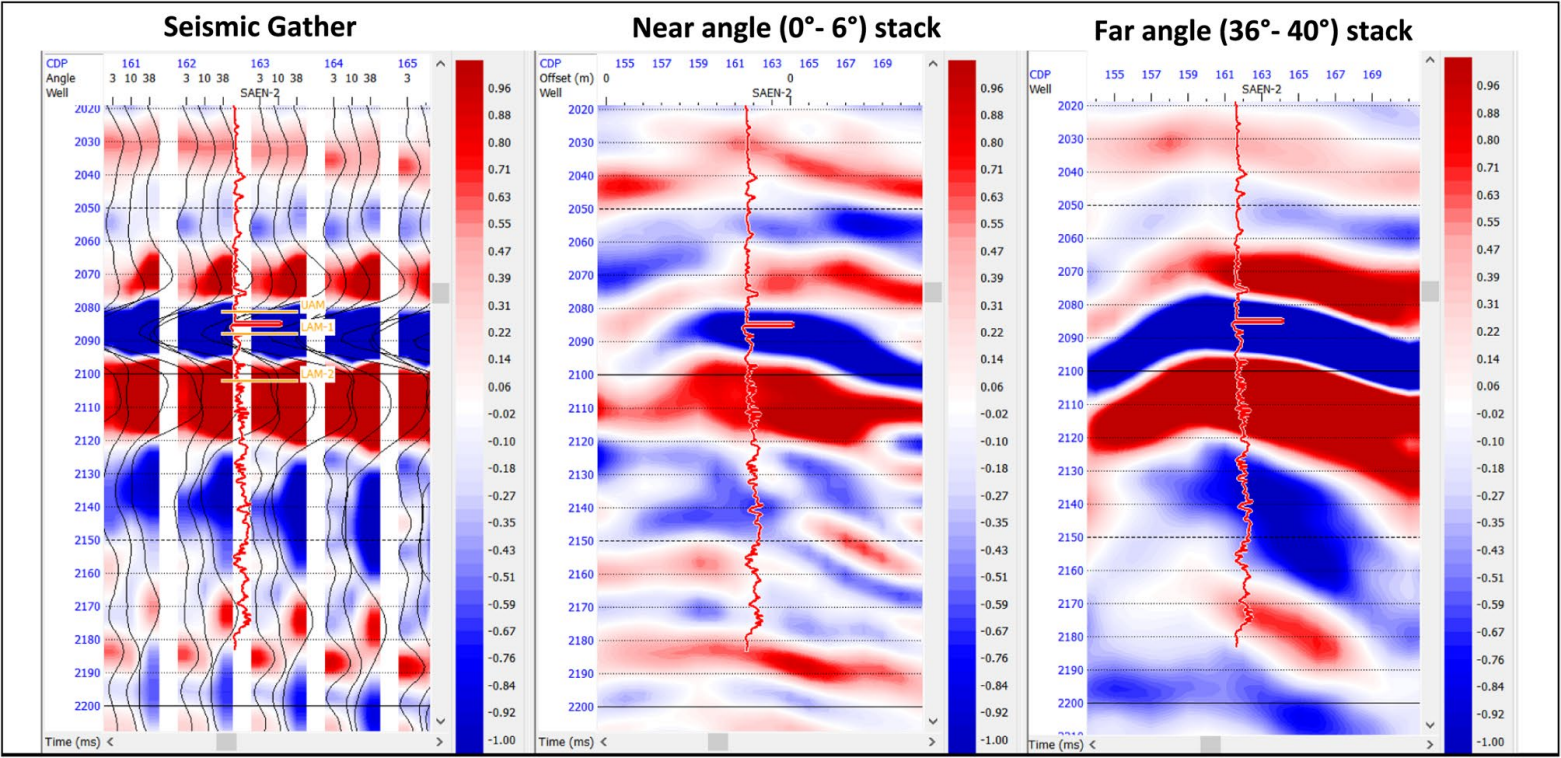
Seismic CMP gathers (**a**), Near angle stack (**b**) and Far angle stack (**c**), to monitor the amplitude variation with different angles at South Abu El Naga Gas Field.

AVO reflectivity is a first step in pre-stack seismic inversion usually used to obtain ordinary reflectivities of P-and S-wave incidence through the assessment of AVO^46^. The AVO method involves analyzing near and far angle stacks, where the amplitude of the “bright-spot” event is stronger on the far-angle stack than it is on the near-angle stack^47^. The amplitude anomaly can be monitored in both stacks (Near and Far), hence helping in providing information about the amplitude behavior with offset. Figure 6 showing Late Miocene Abu Madi low impedance sand, which are characterized by the increase of the negative amplitude from Near to Far seismic angle stacks.

The AVO response of the reflector is can be described by two parameters: the intercept or reflectivity (amplitude) at the zero-offset and the gradient of the amplitude variation with offset. In this study, the intercept (A) and gradient (B) sections have been generated from near, mid, and far angle stacks. AVO attributes, which include the multiplication, summation, and subtraction of the intercept and gradient carried out for Abu Madi sand reservoir. Each of these attributes has significant response for the gas sand.

**Fig. 7:**
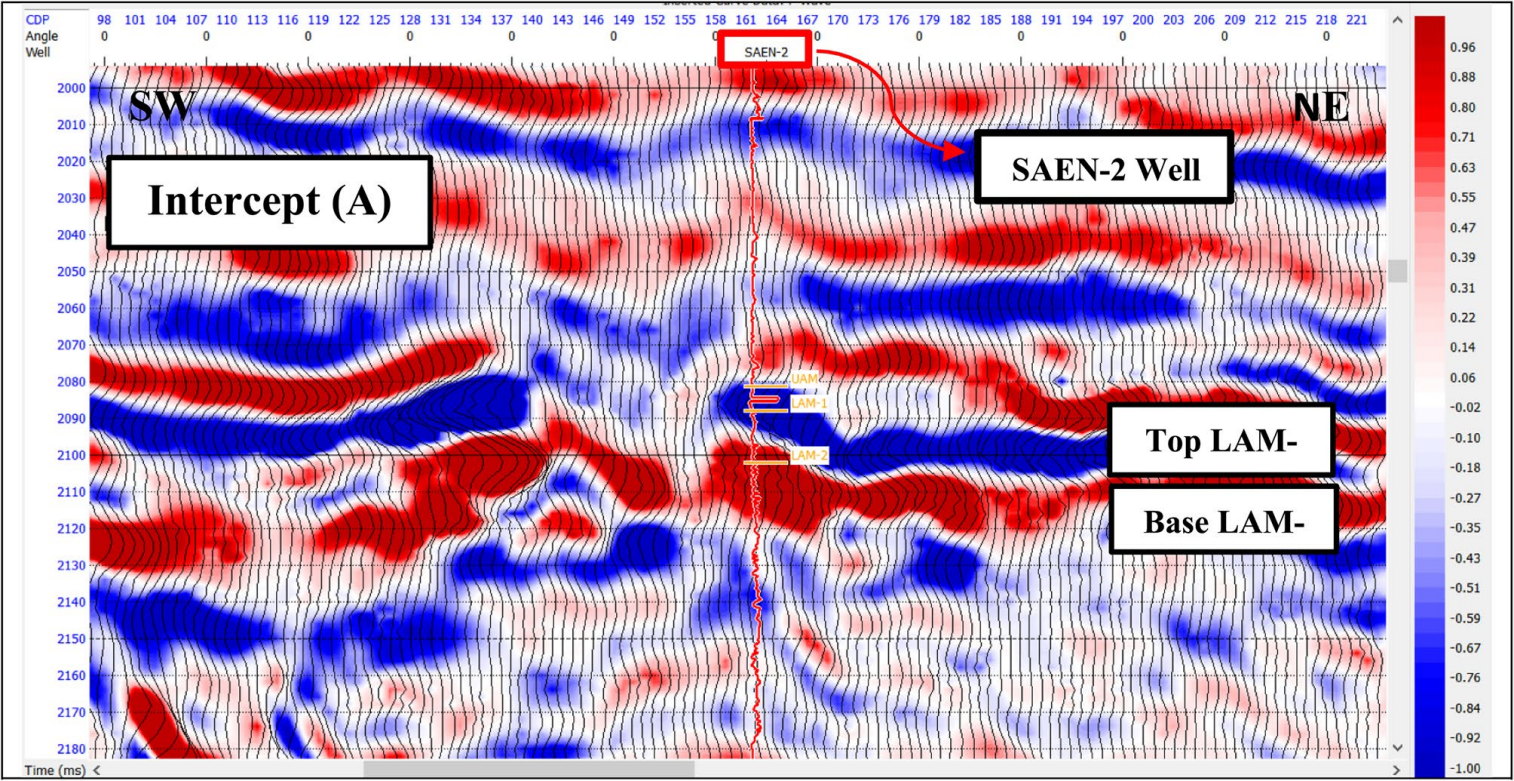
Intercept (A) section passing through SAEN-2 well.

**Fig. 8:**
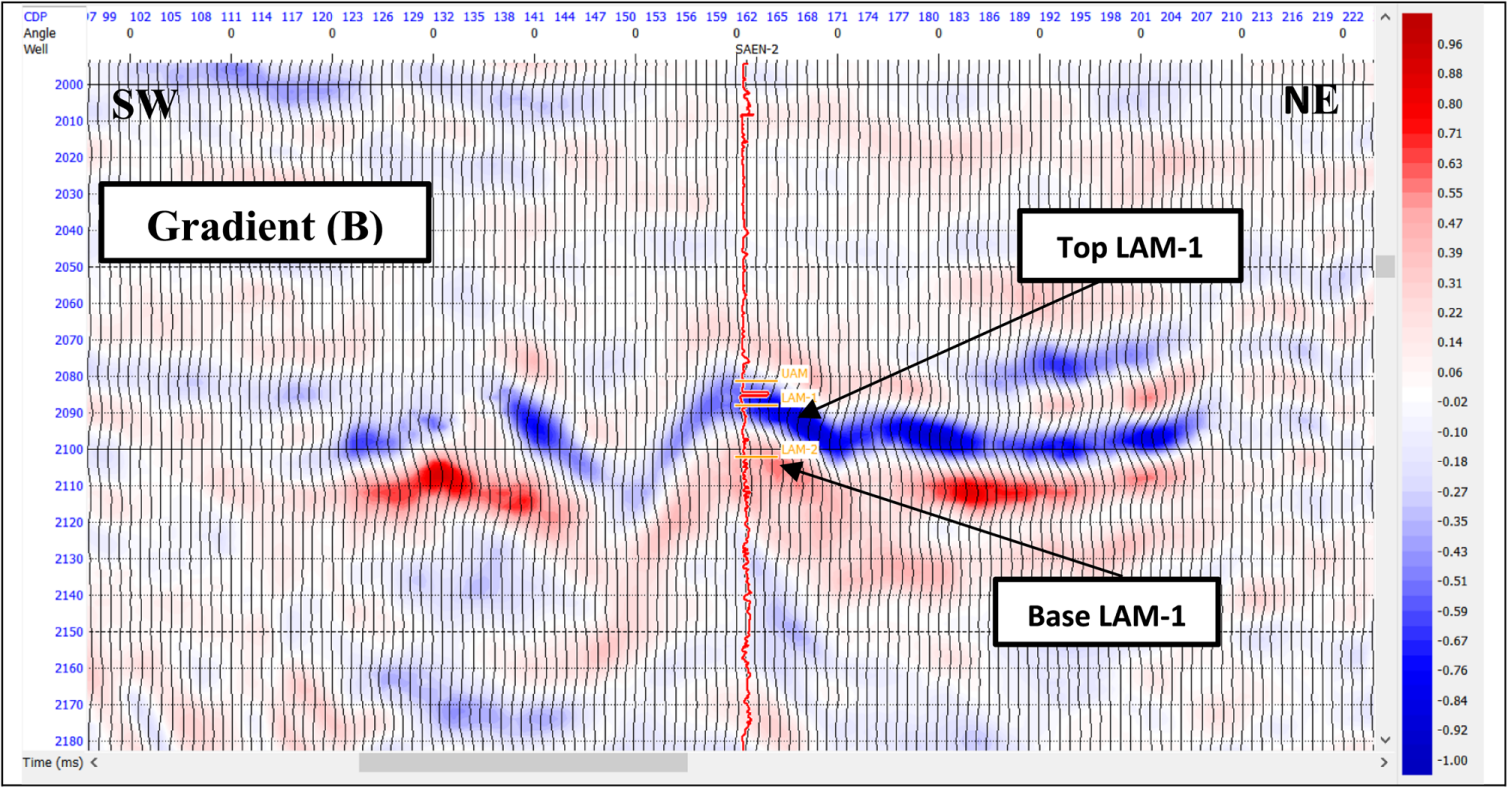
Gradient (B) section passing through SAEN-2 well.

Figure 7 is an intercept A section passing through SAEN-2 well and showing a good separation of the top from the base of Lower Abu Madi-1 with negative (blue trough) amplitude at top and positive (red peak) amplitude at base. While, Fig. 8 is a gradient B section showing strong negative (blue trough) amplitude at top and positive (red peak) amplitude at its base with a better result than intercept A.

**Fig. 9:**
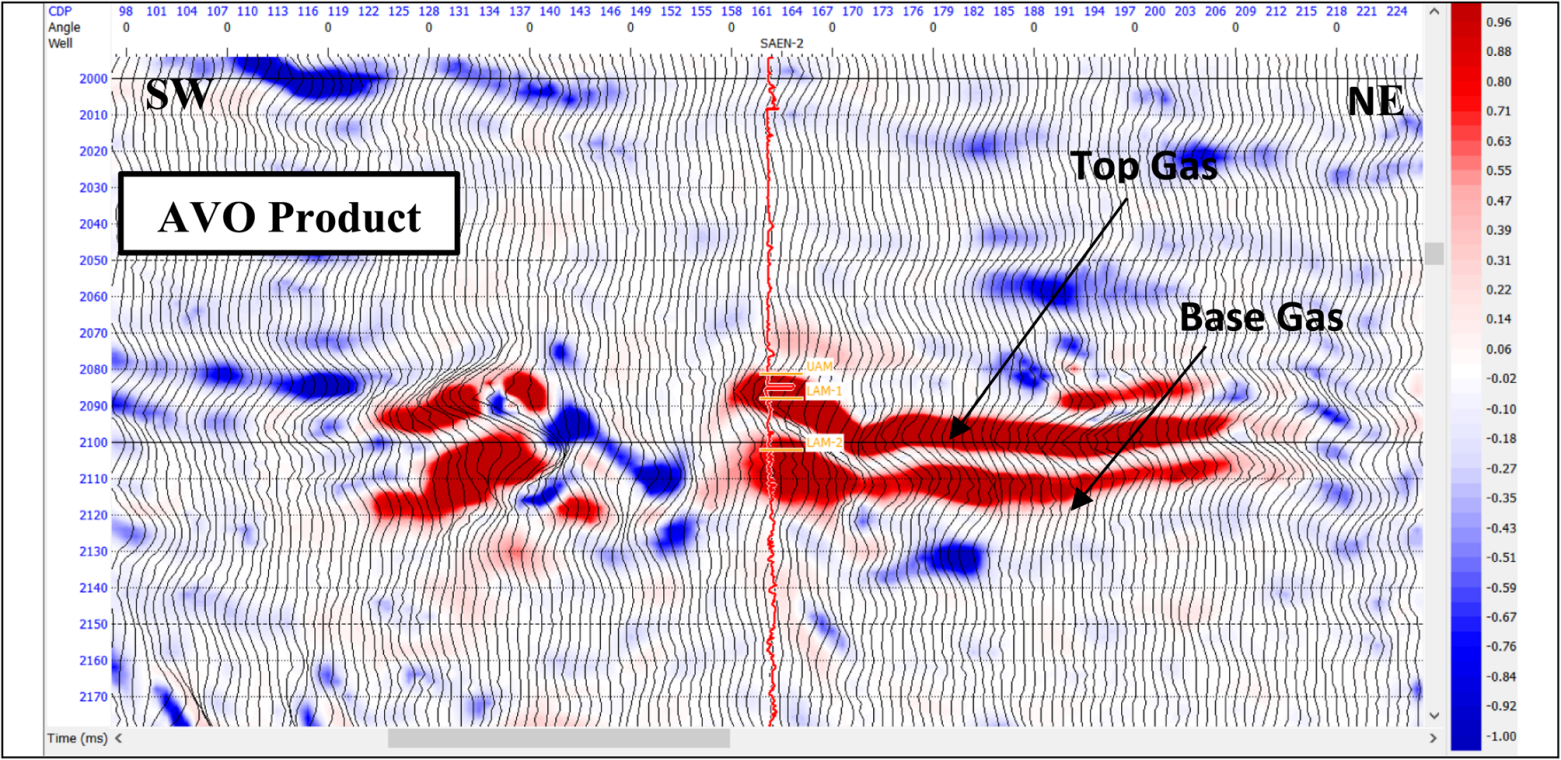
AVO product (A*B) cross section at SAEN-2 well location highlights the AVO anomaly at the gas-bearing sand.

AVO product is a good indicator of the classical bright spots, in which high amplitudes (A) and increased gradients (B) occur simultaneously^48^. Figure 9 is AVO response from the product of the intercept and gradient (A*B) showing strong positive amplitude anomaly at the top of the gas bearing sand zone and strong positive at the base because both A and B are negative at its top and they are positive at the base. This AVO product confirms the presence of class III AVO analysis.

**Fig. 10:**
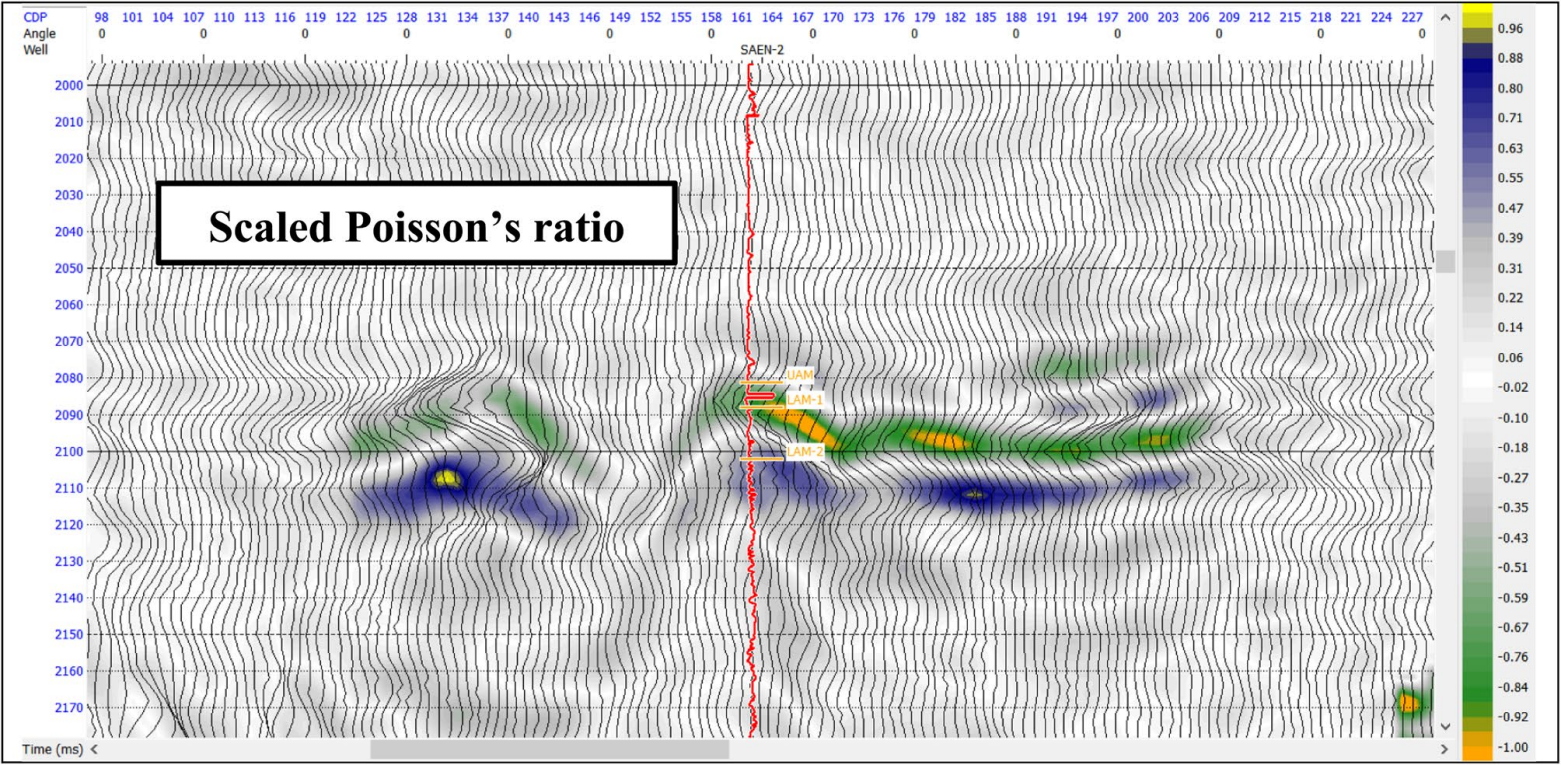
Scaled Poisson’s ratio AVO attribute showing the top as trough and bottom as peak for the gas sand reservoir.

Poisson’s ratio is one of the best indicators for the presence of gas saturated sediments. Scaled Poisson’s ratio AVO attribute (A + B) shows variation based on the fluid content of the reservoir. The extraction of this attribute is considered the best way to show the AVO anomaly using the color data show the scaled Poisson’s ratio values at the two-way time between 2090 and 2100 ms as shown in Fig. 10. This attribute shows a very low value at the top of gas sand reservoir zone with higher values at its base. By calibration of the change in Poisson’s ratio with resistivity, Vp, and gamma ray logs confirms that the low scaled Poisson’s ratio values and the gas saturated sand are good matched.

2.B) Impedance methods.

**Fig. 11:**
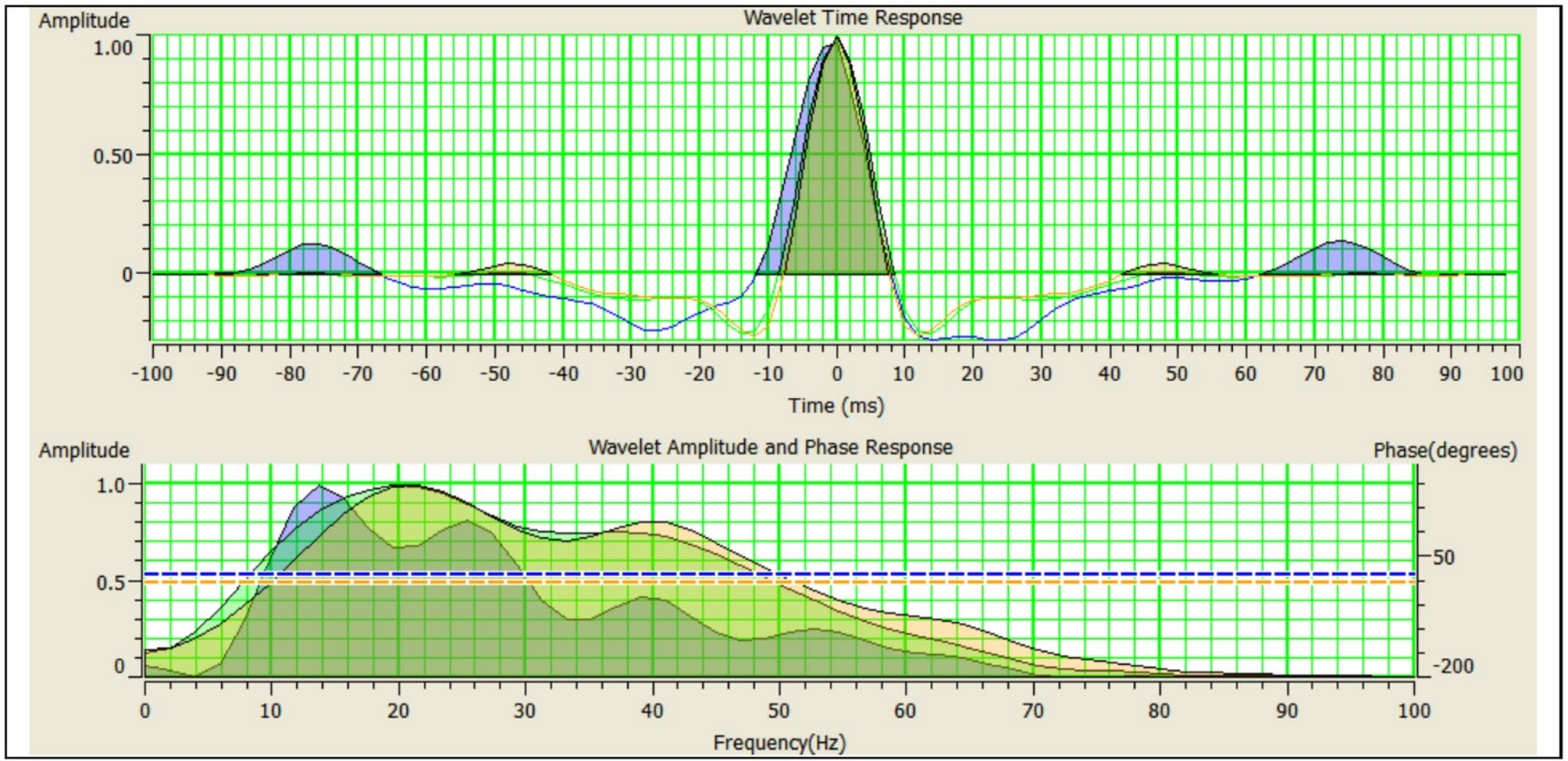
Wavelets extracted from angle stacks gives better matching.

Pre-stack inversion uses the partial angle stacks of near (0°–6°), mid (8°–12°) and far (36°–40°) of our available seismic data, that were converted to angle gathers shown in Fig. 4. The pre-stack inversion extracts P-impedance (Zp), S-impedance (Zs), and elastic impedance (EI). Consequently, Vp/Vs ratio as well as LMR (lambda-Mu-rho) analysis can be estimated in terms of λρ and μρ. That is to detect gas sand channel compartments within Abu Madi Formation in South Abu El Naga field. The simultaneous pre-stack inversion started with the building of the initial models of Zp, Zs, and ρ which were further interpolated between the wells using horizons as the structure guides for the interpolation. Then, model-based synthetic traces are created with the help of the three extracted wavelets for near (orange), mid (green) and far (blue) shown in Fig. 11. To improve the fit between the actual seismic traces and the synthetic traces, it iteratively modified the initial models.

**Fig. 12:**
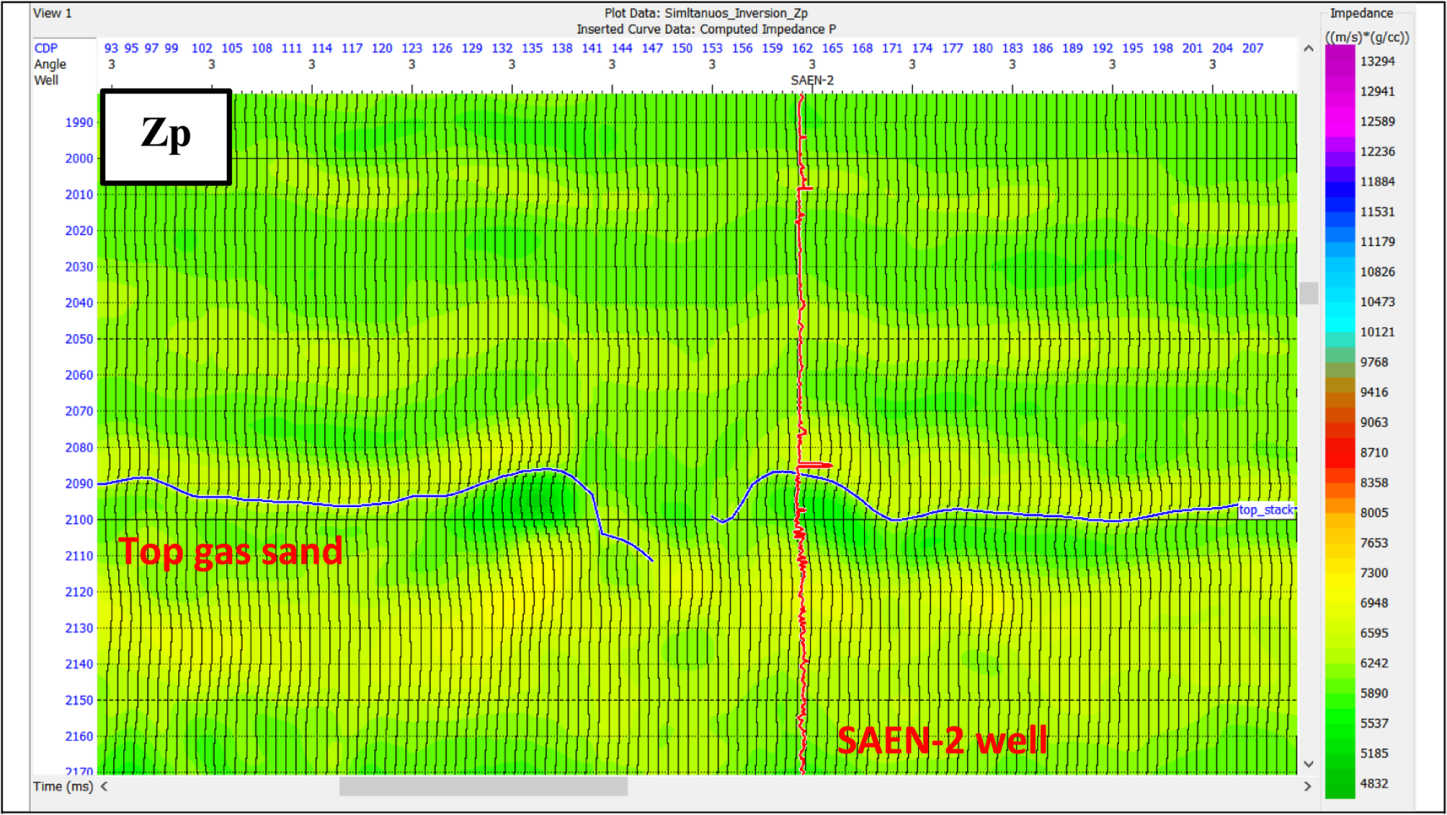
Zp indicates low impedance of gas reservoir zone in dark green color.

Simultaneous pre-stack inversion depends on the relationship between ln(Zp), ln(Zs), and ln(ρ) at the well locations, from which the coefficients (k, kc, m and mc) are calculated. Deviations of these values from the background ΔL_S_ and ΔL_D_ were calculated from the inversion itself. The result of pre-stack is the P-impedance, S-impedance, density and Vp/Vs ratio. The P-impedance and Vp gave information on the nature of the lithology and fluid as shown in Fig. 12.

**Fig. 13:**
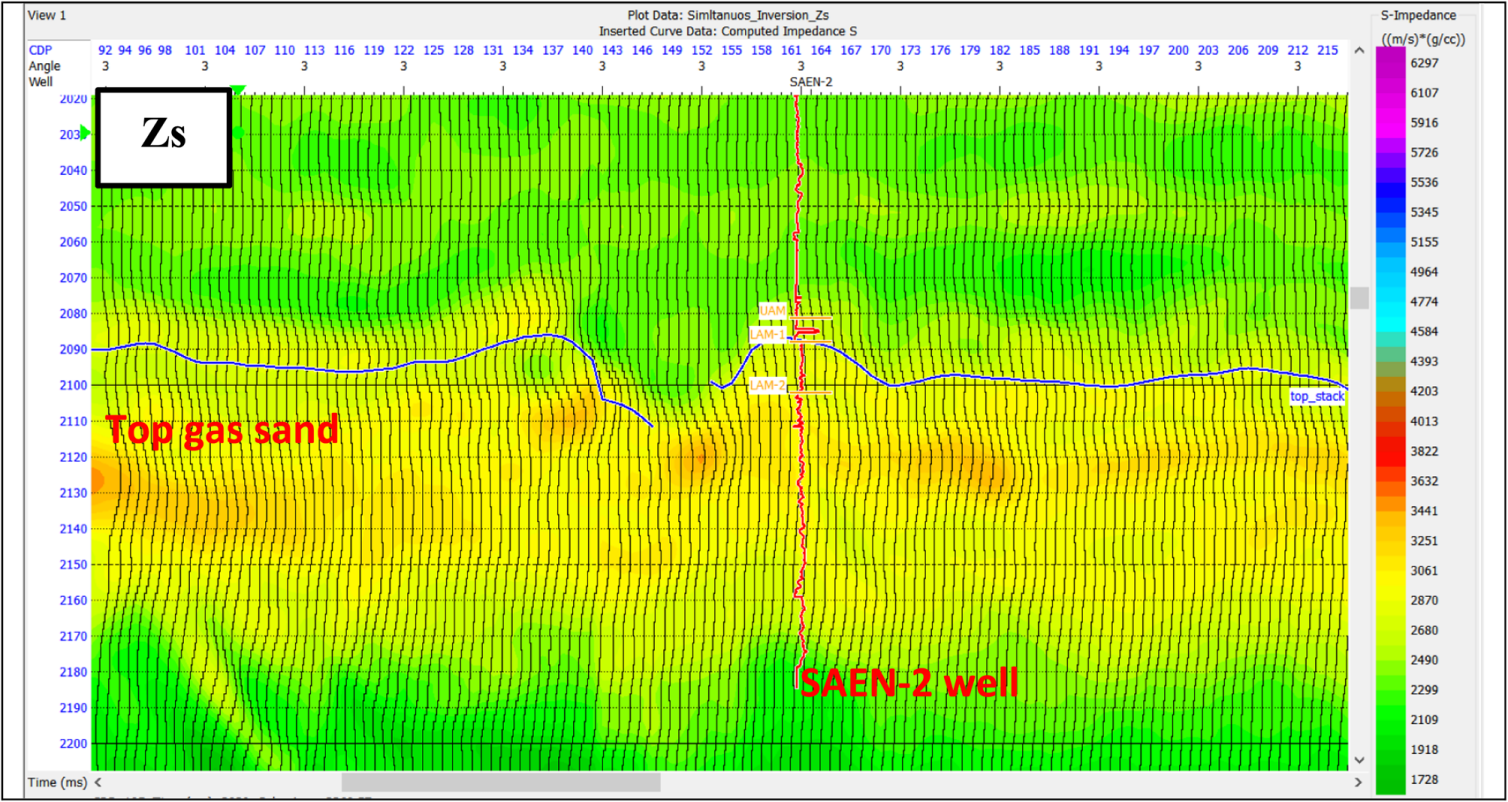
Zs indicates the relatively high of gas reservoir zone in red color.

The S-impedance and Vs are considered as a lithological indicator, because S-wave cannot pass through liquids as shown in Fig. 13. The S-wave impedance shows relatively high impedance compared to the P-wave impedance, because the shear wave velocity slightly increases within a hydrocarbon reservoir, unlike the compressional wave, whose velocity decreases dramatically within a gas reservoir^49^.

**Fig. 14:**
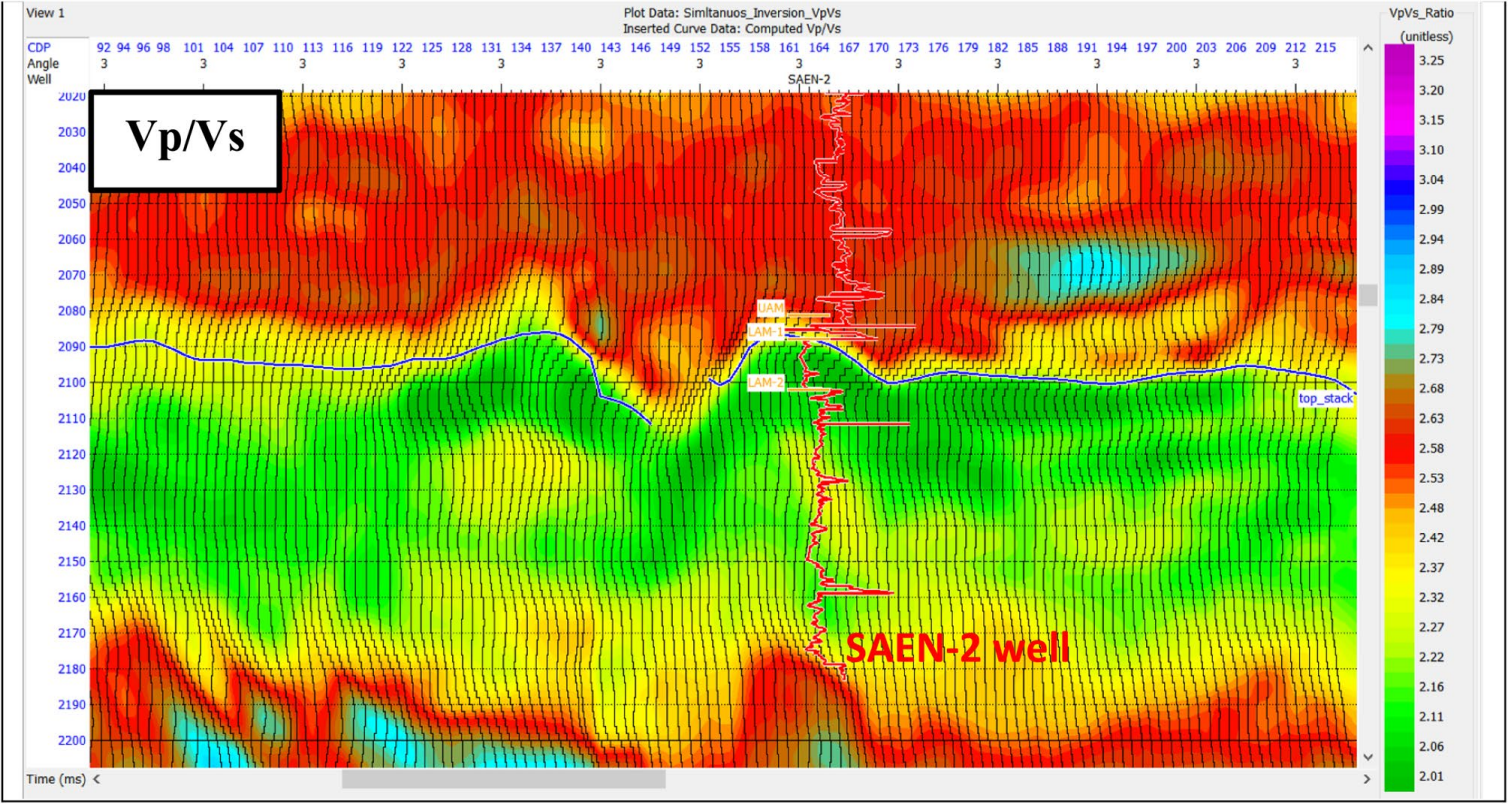
Vp/Vs ratio indicates a significant decrease (green) in gas sand reservoir zone.

The Vp/Vs ratio is an effective lithology indicator because each lithology exhibits a defined trend that is independent of porosity and depth. However, the Vp/Vs ratio is affected by formation anisotropy, the ratio values may not be absolute indicators of a particular lithology. Notice the low ratio at the gas sand as shown in Fig. 14.

**Fig. 15:**
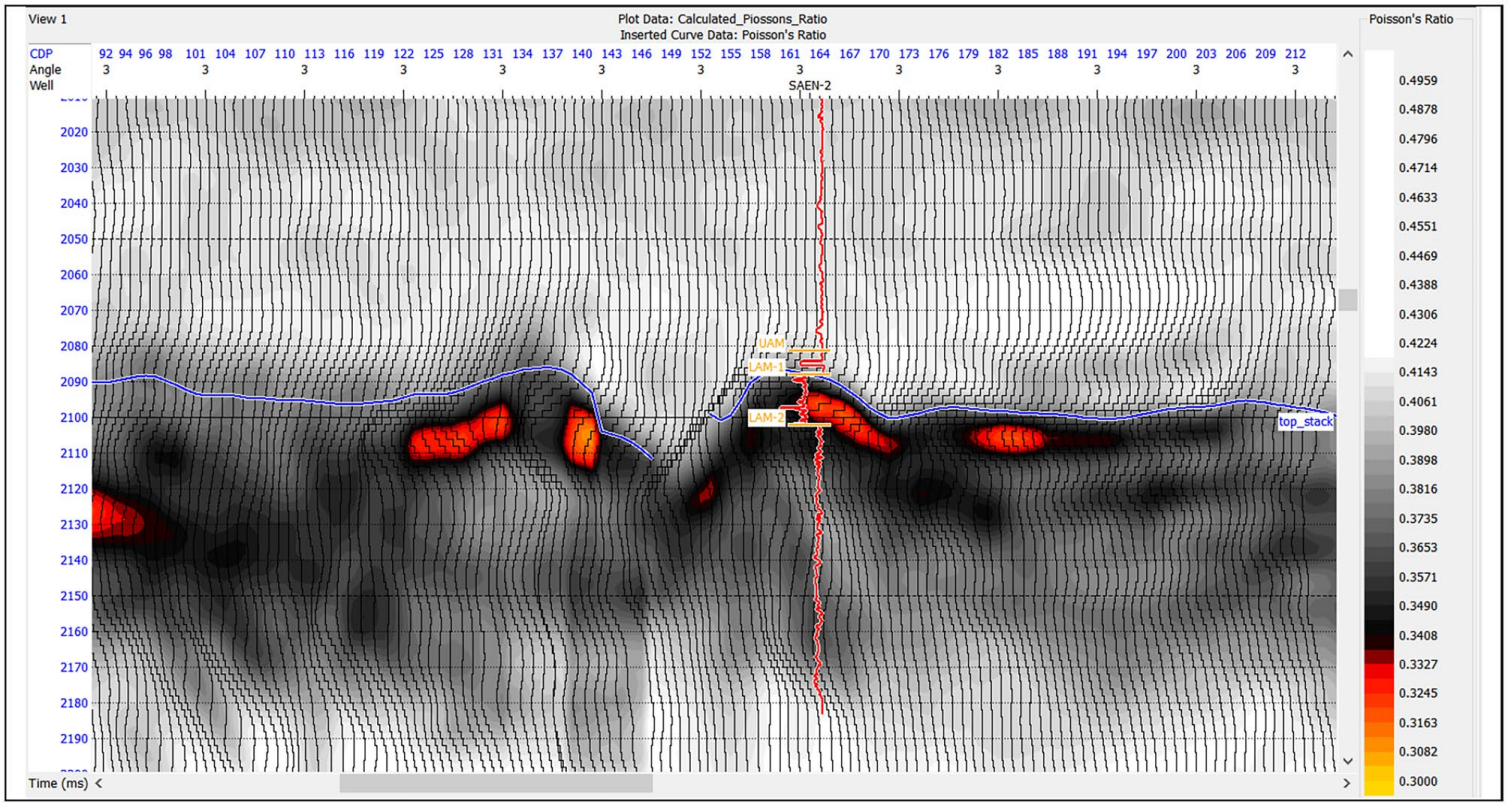
Poisson’s ratio section indicates lower values in gas reservoir zone (red).

The Poisson’s ratio is an important parameter for exploration of oil and gas. It can be used to detect seismic expressions associated with hydrocarbon. The Poisson’s ratio is sensitive to changes in the pore fluid, and it can be estimated from compressional and shear wave velocities. Gas-saturated rocks tend to have lower Poisson’s ratios than liquid-saturated rocks, because gas is more compressible than liquid. Therefore, by measuring the Poisson’s ratio of a rock layer, one can infer the presence or absence of gas in the pores as shown in Fig. 15.

3. Lambda Mu Rho analysis.

**Fig. 16:**
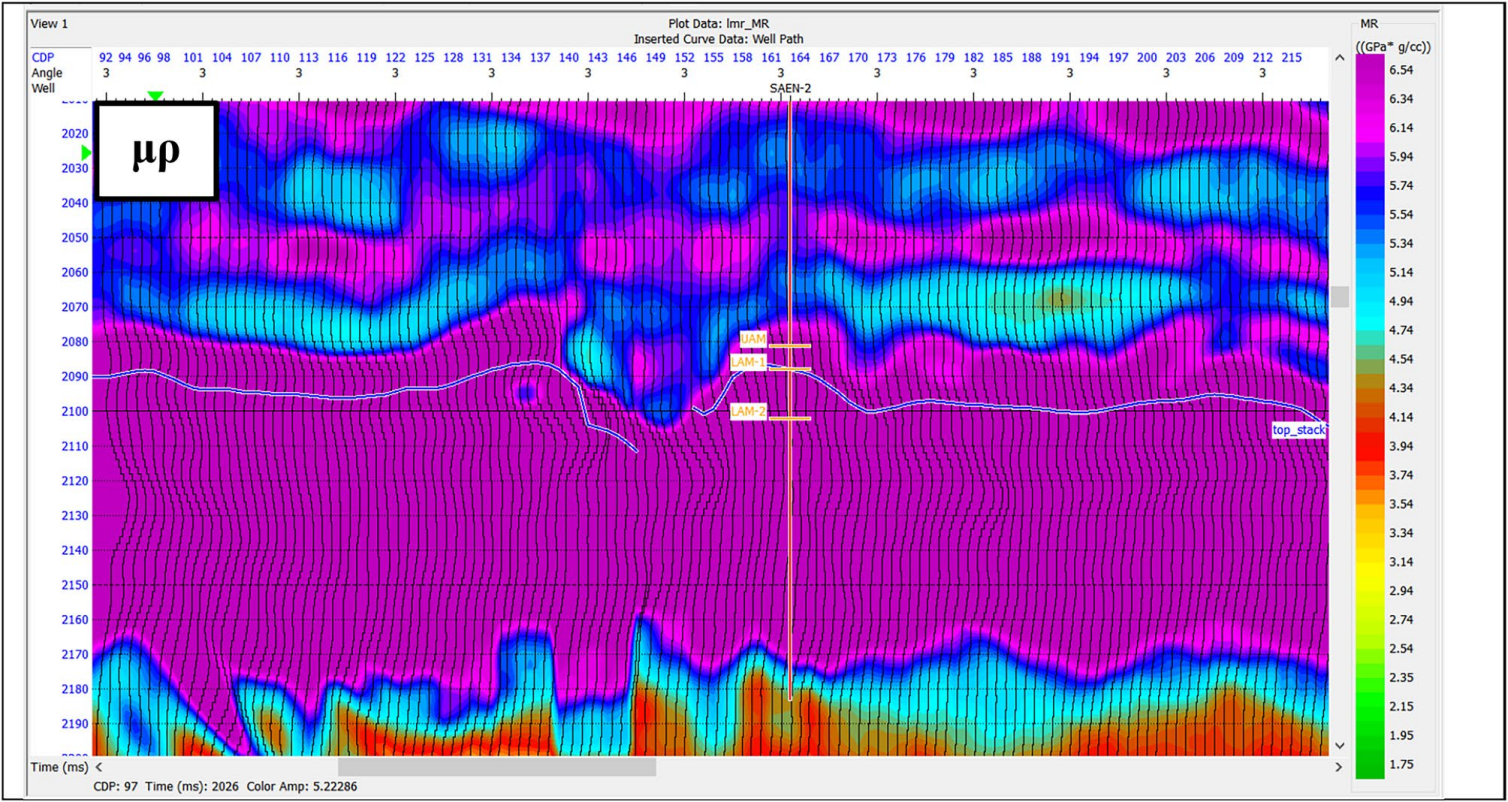
The μρ section with high values as it is a lithology indicator.

**Fig. 17:**
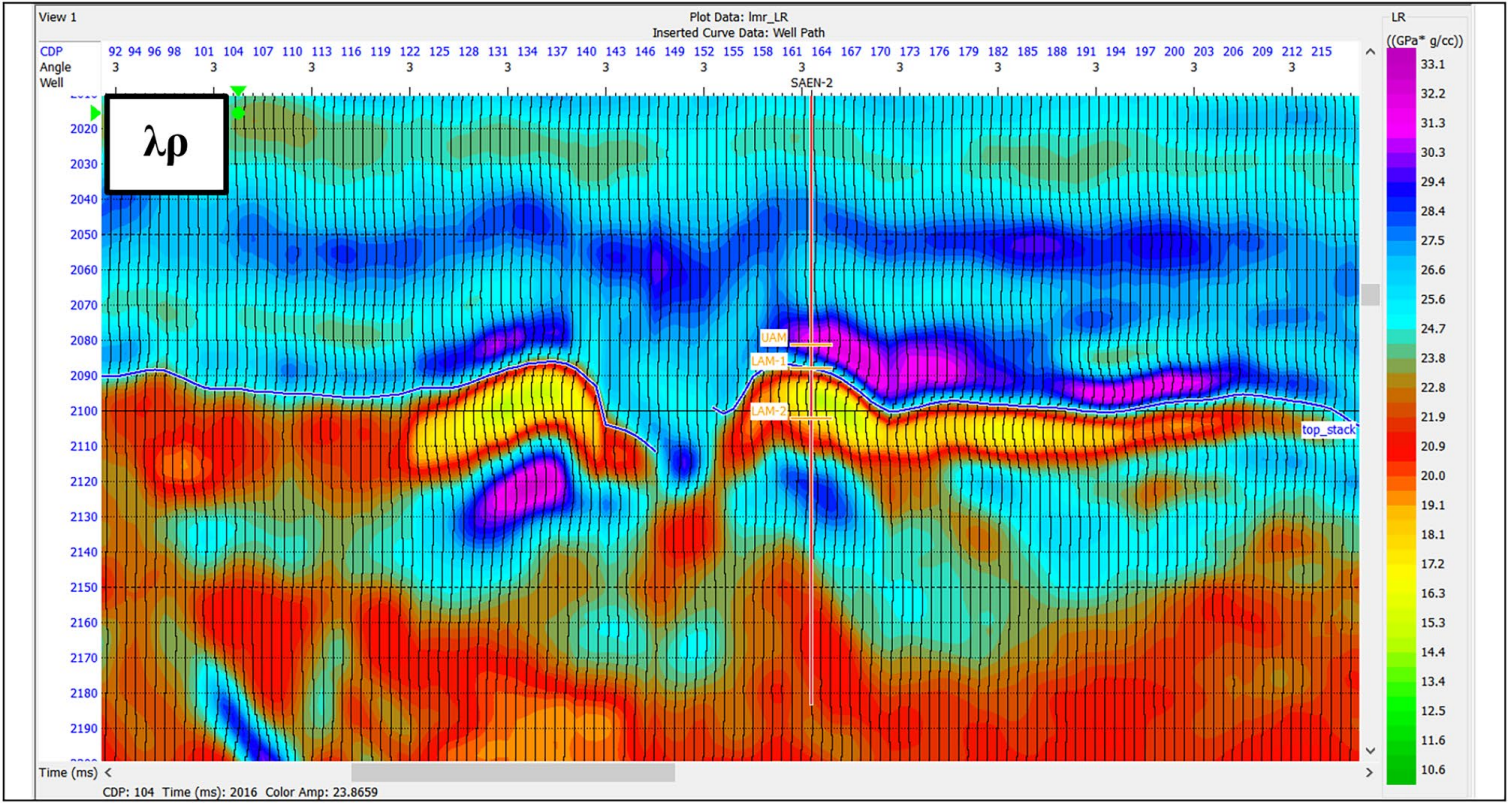
The λρ attribute with lower values at gas sand zone.

The Lambda-Mu-Rho attributes (LMR) are defined so that the Lame’s elastic parameters λ and μ are combined with density ρ in the form of λρ (Lambda Rho) and μρ (Mu Rho), as was first proposed by Goodway et al.^43^. LMR (λ, μ and ρ) and the ratio of the compressional-wave to shear-wave velocities (VP/VS) are useful attributes that can be extracted from the results of simultaneous inversion. Pre-stack seismic CMP gathers are inverted to extract the P-impedance and S-impedance, and from these impedances, the λρ and μρ products are extracted. Products of λρ (referred to as the “LR” attribute) and μρ (“MR” attribute) are also extracted from the impedances. Figure 16 shows μρ section indicating high values as it is sensitive to lithology more than fluid content, while. λρ indicates lower values as it is sensitive to a fluid content than lithology discrimination as shown in Fig. 17.

## Discussion

Integrating different techniques provide more details and help in understanding the study area. The results were consistent with seismic inversion, which helped in the interpretation of the reservoir and confirmed that gas sand in the study area is characterized by a low impedance which aligns with bright amplitude anomalies in the seismic, supporting their interpretation as gas sand reservoir. The AVO analysis revealed distinct amplitude variations with offset across multiple seismic gathers in the target interval, interpreted as potential gas-bearing zones. The inversion sections show improved lateral lithology changes and discontinuities, enhancing mapping of potential compartment boundaries. These consistent indicators across all AVO attributes strongly indicate the presence of gas sands within the Upper Miocene Abu Madi Formation.

The use of limited well logs and 2D seismic data in subsurface interpretation presents several significant challenges. With limited well data, the uncertainty in subsurface predictions increases, poor stratigraphic correlation and key reservoir characteristics, such as porosity, lithology, and fluid saturation, become difficult to map which led to low petrophysical accuracy^50^. Additionally, 2D seismic data provides only a limited view of the subsurface, typically along a single line or cross-section, which reduces the ability to fully capture lateral variations in geology. This can lead to high risk of structural misinterpretation of subsurface features, as lateral continuity and fault positioning. Additionally, the interpretation of 2D seismic data is more ambiguities, especially in areas with complex or heterogeneous geology, where key structural or stratigraphic features may not be captured along the survey lines^51^. Noise and multiple energy can distort amplitude variation with offset, leading to false anomalies. Lateral changes in lithology and compaction can mask fluid effects, reducing the reliability of impedance-based discrimination.

The study introduces the use of Amplitude Variation with Offset (AVO) reflectivity attributes, impedance methods, and lambda-mu-rho (LMR) as an innovative approach to improve gas sand detection under such seismic and well data limitations. The study successfully delineates the reservoir and enhances the interpretation of gas-bearing zone. The most significant findings include the identification of anomalous AVO responses correlating with potential pay zones and this approach can be used for similar geologically complex or data constrained settings.

## Conclusions

Pre-stack seismic inversion is an effective method for detecting Late Miocene (Messinian) gas-bearing sandstone reservoir in Lower Abu Madi Formation at South Abu El Naga gas field in West El Manzala concession, Onshore East Nile Delta, Egypt, which is the main aim of the study. AVO reflectivity attributes and pre-stack impedance attributes provide excellent results for gas bearing clastic reservoirs delineation. The LMR attributes λρ and μρ are related to the mechanical properties of rocks. These attributes are usually used to describe the properties of rock matrix and pore fluid.

AVO reflectivity attributes such as intercept, gradient, AVO product and scaled Piosson’s ratio have been estimated for gas channel identification, where the gradient B attribute section shows very strong negative amplitude at the top of Lower Abu Madi Formation gas reservoir with a better result than intercept A. Moreover, AVO product was a good indicator showing strong positive amplitude anomaly at the top of the gas bearing sand zone. Low values of scaled Poisson’s ratio due to the presence of fluid content show another good direct indicator of gas reservoir.

At the top of gas sand reservoir zone, Zp, Vp, Vp/Vs ratio, λρ and σ show lower values, while Zs and μρ show higher values. Obviously, Zp, Vp, Vp/Vs and λρ results are fluid sensitive and used as gas sand indicators, while Zs, μρ and density values are used for lithology separation purposes.

The integration of these three techniques; AVO reflectivity attributes and pre-stack seismic impedance inversion and LMR analysis indicate that Abu Madi gas sand channel reservoir is divided into two separate flanks crossed by mud filled cutting channel which acts as a lateral seal for these two compartments.

## Data Availability

The raw data used in the current study remains confidential to the Egyptian General Petroleum Corporation (EGPC) and used with permission and is not publicly available. Data was available to authors with permission from EGPC (the Egyptian General Petroleum Corporation), EGPC email: EGPC@egpc.com.eg.
