# Correction: AVO reflectivity and pre-stack seismic impedance inversion for gas sand channel detection at South Abu El Naga Field, Onshore Nile Delta, Egypt

**DOI:** 10.1038/s41598-025-27982-y

**Published:** 2025-11-28

**Authors:** Nourhan Ahmed Al-Ashqar, Abdel-Khalek El-Werr, Ahmad Sobhy Helaly, Azza Kamel

**Affiliations:** 1 Geophysics Department, Faculty of Science, Ain Shams University, Cairo, Egypt; 2 Applied Geophysics (Seismic Methods), Geophysics Department, Faculty of Science, Ain Shams University, Cairo, Egypt; 3 Applied Geophysics (Potential Methods), Geophysics Department, Faculty of Science, Ain Shams University, Cairo, Egypt; 4 Exploration and Board Member, El-Wastani Petroleum Company (WASCO), Plot No. 188, El Tesaeen St., Fifth Settlement, New Cairo, Egypt

Correction to: *Scientific Reports* 10.1038/s41598-025-04251-6, published online 20 June 2025

The original version of this Article contained errors in Fig. 1, where boundaries and borders in the location map of the study area were represented incorrectly. The original Fig. 1 and accompanying legend appear below. 

**Fig. 1:**
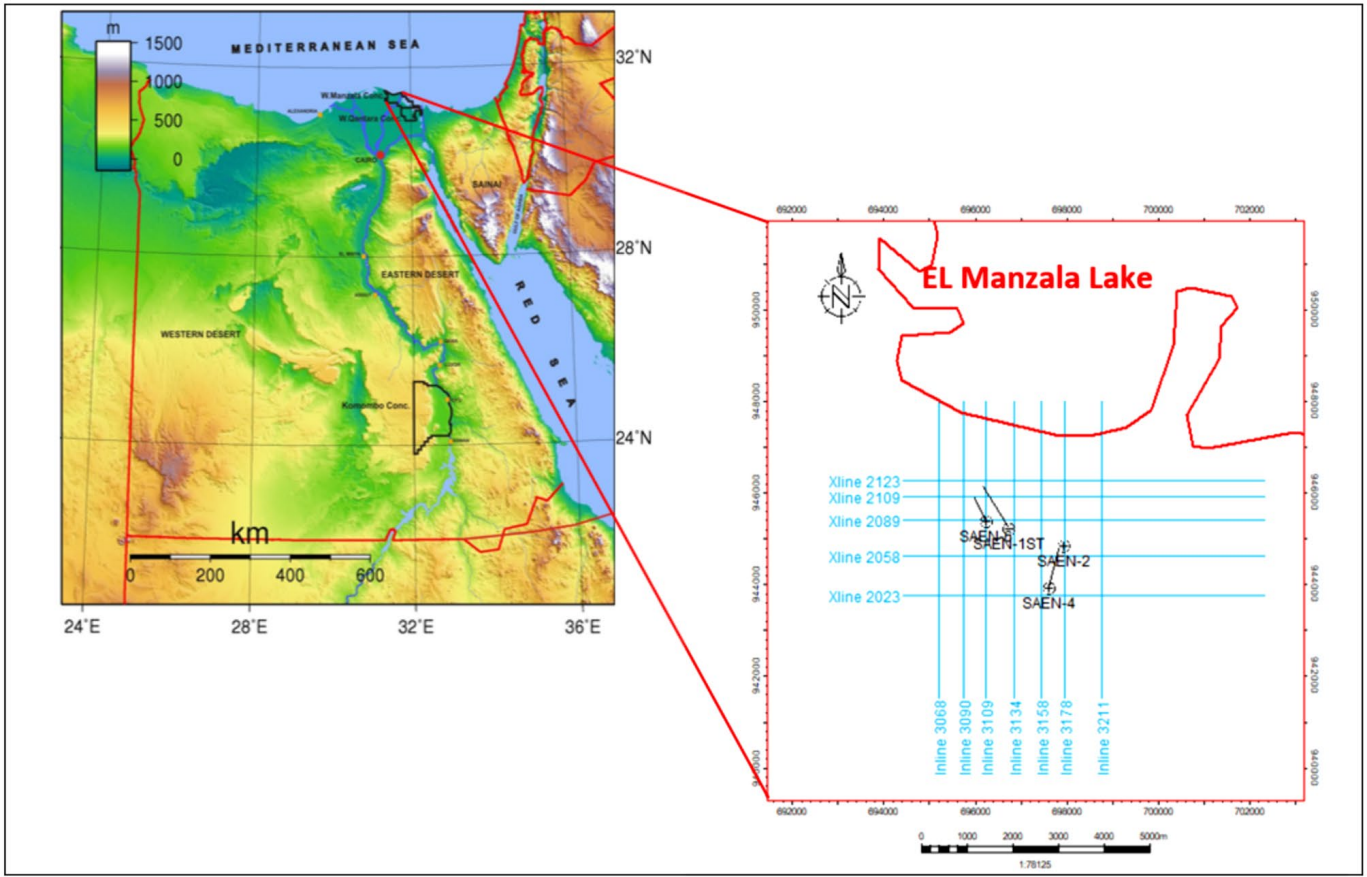
Location map of the West El Manzala concession study area and Base map of the four wells and the 2D-seismic lines, created using Petrel (2019) software, Petrel subsurface software | SLB.

The original Article has been corrected.

